# Carpal tunnel syndrome in mucopolysaccharidosis type I: clinical, surgical and histopathological findings

**DOI:** 10.1177/17531934261416366

**Published:** 2026-02-08

**Authors:** Boudewijn AW van Binsbergen, Joris A van Dongen, Linda Vriend, Stephan H Goedee, Peter M van Hasselt, Aebele B Mink van der Molen

**Affiliations:** 1 Department of Plastic Reconstructive and Hand Surgery, University Medical Center Utrecht, Wilhelmina Children’s Hospital, The Netherlands; 2 Doctors At Soap, Amsterdam North-Holland, The Netherlands; 3 Brain Center, Department of Neurology and Neurosurgery, University Medical Center Utrecht, The Netherlands; 4 Department of Pediatric Metabolic Diseases, University Medical Center Utrecht, Wilhelmina Children’s Hospital, The Netherlands

**Keywords:** Carpal tunnel syndrome, compression neuropathy, haematopoietic stem cell transplantation, mucopolysaccharidosis type I, pediatric, surgery

## Abstract

**Introduction:**

Mucopolysaccharidosis type I is a rare metabolic disorder characterized by the accumulation of glycosaminoglycans, leading to musculoskeletal disorders such as carpal tunnel syndrome. This retrospective cohort study was performed to determine the prevalence and timing of the development of carpal tunnel syndrome, assess the efficacy of surgery on recurrence and the effect of haematopoietic stem cell transplantation in resolving accumulated degradation products.

**Methods:**

Thirty-three mucopolysaccharidosis type I patients, born between 2001 and 2023, were included in this study. They received annual screening and treatment at our specialist hospital. Patient demographics, clinical symptoms of carpal tunnel syndrome pre- and post-carpal tunnel release and histopathological sections of the flexor retinaculum were collected. Regression analyses were conducted to assess cumulative risks and determine hazard ratios and survival curves were plotted.

**Results:**

Twenty-seven of 33 mucopolysaccharidosis type I patients, with a median age at the latest follow-up of 13 years (IQR 7.7 to 18.3), were diagnosed with carpal tunnel syndrome on electrophysiological tests or ultrasound at a median age of 3.6 years (IQR 2.8 to 5.2). Only a few patients exhibited clinical symptoms. Twenty-one patients underwent carpal tunnel release and 13 patients experienced recurrence. The highest risk of developing carpal tunnel syndrome was within the first 6 years of life. Eight of the 11 patients accumulated degradation products within lysosomes despite successful haematopoietic stem cell transplantation.

**Conclusions:**

In this study, 27 mucopolysaccharidosis type I patients were diagnosed with carpal tunnel syndrome, showing a high-risk window from 0 to 6 years of age. Recurrence of carpal tunnel syndrome after carpal tunnel release surgery was common, occurring in 61.9% of patients.

**Level of evidence:**

III

## Introduction

Mucopolysaccharidosis type I (MPS-I) is a rare lysosomal storage disorder. It can be divided into three subcategories based on the phenotype. From most to least severe these are: Hurler, Hurler–Scheie and Scheie. The incidence of MPS-I is approximately one patient per 100,000 live births (Matalon et al., 2025). The *a*-l-iduronidase (IDUA) gene encodes the IDUA enzyme. In MPS-I, a mutation in the IDUA gene results in IDUA enzyme deficiency. The IDUA enzyme is required for the degradation of the glycosaminoglycans (GAGs) dermatan sulfate and heparan sulfate (Bio-Techne, 2025). Deficiency of this enzyme leads to the accumulation of partially degraded GAGs in lysosomes and the extracellular matrix of several tissues such as bone, brain and connective tissue. This accumulation results in multisystem disease manifestations such as progressive neurocognitive impairment and a broad spectrum of musculoskeletal disorders (Parini et al., 2017).

Different treatment modalities exist for MPS-I, including haematopoietic stem cell transplantation (HSCT) and enzyme replacement therapy (ERT). Since 1981, severely affected MPS-I patients have been treated with HSCT and since 2003, more attenuated MPS-I patients have been treated with ERT (Clarke, 2024; Guffon et al., 2021; Hobbs et al., 1981; Miebach, 2005). These treatment modalities have been shown to alleviate disease manifestations; however, they appear insufficient in preventing or resolving all symptoms, especially musculoskeletal manifestations such as carpal tunnel syndrome (CTS) (Alonzo-Rojo et al., 2017; Beck et al., 2014; Field et al., 1994; Haddad et al., 1998; van Heest et al., 1998; Weisstein et al., 2004). The pathophysiology of CTS in MPS-I is not well understood. It is rare in healthy children but is frequently observed in patients with MPS-I. The hypothesis for the development of CTS in children with MPS-I is the accumulation of GAGs within the connective tissues of the hands as well as skeletal abnormalities (dysostosis multiplex). Surgical release of the carpal tunnel is the treatment of choice for CTS. Despite successful carpal tunnel release (CTR), recurrence is frequently observed in MPS-I patients (Thirache et al., 2022; Viskochil et al., 2017).

This retrospective study aimed to improve the understanding of the pathophysiology of CTS in MPS-I by examining the effect of HSCT in resolving the accumulation of degradation products and assessing the prevalence, timing of development and the efficacy of surgery on the recurrence of CTS.

## Methods

### Study design and population

A retrospective cohort study was carried out adhering to the STROBE guidelines. All patients received HSCT at our specialist hospital. After transplantation, the care of these patients was centralized at our specialized centre for follow-up after HSCT and all patients received annual full-body screenings.

The study protocol was approved by the Institutional Review Board. This study was carried out in accordance with the Helsinki Declaration of 1975, revised in 2008. Written informed consent was obtained from all patients and/or their legal representatives.

### Baseline characteristics of MPS-I patients

This study retrospectively collected data from patients’ medical records. The data included patient demographics (age and sex), age at the time of HSCT (months), phenotype and mutation traits, biochemical data (urinary heparan and dermatan sulfate concentrations (µg/mmol creatinine)) and IDUA enzyme levels ((µmol/h/L blood) after successful HSCT), and follow-up period. Successful HSCT was defined as the achievement of full donor chimerism. The follow-up period was defined as the time between the date of HSCT and the date of the last visit. Furthermore, the characteristics of CTS (age at diagnosis and recurrence, electrophysiological test and ultrasound (US) values) and CTR (age at surgery) were collected. To analyse group characteristics, means and medians with interquartile ranges were used.

### Examination of the hand of patients with MPS-I and carpal tunnel syndrome

This screening included hand examination by a metabolic paediatrician, paediatric neurologist and paediatric physiotherapist. Electrophysiological tests and US examinations were routinely performed to assess CTS. Electrophysiological tests were considered positive for CTS if both motor and sensory latency differences were prolonged in a single hand, that is, >0.4 ms. Ultrasound measured the transverse cross-sectional area of the median nerve at the distal wrist crease and at one-third of the forearm. Carpal tunnel syndrome was considered positive if the cross-sectional area of the median nerve at the carpal tunnel and/or forearm was greater than 3.9/4.0 mm^2^ (age 0–3 years), 4.7/5.6 mm^2^ (age 4–6 years), 5.1/6.2 mm^2^ (age 7–11 years) and 6.7/9.1 mm^2^ (12–16 years), respectively (Cartwright et al., 2012). Carpal tunnel syndrome was diagnosed when either electrophysiological or US measurements met the predefined abnormal thresholds. The time to initial CTS onset was defined as the interval from birth to the first diagnosis of CTS. The time to recurrence was defined as the interval from the date of CTR to the date of recurrent CTS diagnosis.

### Early indicators and risk analyses for development of carpal tunnel syndrome in patients with MPS-I

Four continuous indicators of CTS development were analysed: age at successful HSCT, IDUA enzyme level and urinary heparan and dermatan sulfate concentrations. The outcome of CTS was binary (0: no CTS; 1: CTS). Hazard ratios (HR) and confidence intervals (CI) were calculated using Cox regression analysis. The mean lower and upper limits of normal were calculated for IDUA enzyme levels and urinary heparan and dermatan sulfate concentrations. The cumulative risk for CTS development and recurrence was analysed by carrying out a one minus cumulative survival analysis. All outcomes were analysed at the patient level and bilateral findings did not contribute to additional events. Statistical significance was set at *p* < 0.05.

### Efficacy of carpal tunnel release in MPS-I patients

Open CTR was performed under loupe magnification. Two weeks after surgery, the clinical effects were observed by a paediatric plastic surgeon. A second consultation was performed 2–3 months after surgery by an experienced hand team, consisting of a plastic surgeon specialized in hand surgery, a dedicated hand therapist and a paediatric rehabilitation doctor experienced in hand disorders.

The sensory and motor latency values and conduction velocity values after CTR were compared with the normal values published by Ryan et al. (2019). Carpal tunnel syndrome was classified as persistent when symptoms were still apparent and either electrophysiological tests or US values had not improved at the next consultation. Recurrence was defined as either new abnormal electrophysiological or US findings, based on which a diagnosis of CTS could be made after previously documented postoperative improvement.

For patients who did not undergo CTR, a structured observation protocol was used. This included repeated electrophysiological and ultrasonographic assessments at intervals determined by symptom burden, caregiver concerns and prior test results. The decision for observation rather than immediate surgery was based on clinical judgement, weighing factors such as overall clinical status, the (absence of) clinical symptoms and parental preference.

### Histological analysis of flexor retinaculum tissue

Flexor retinaculum tissue samples (*n* = 11) were obtained on an opportunistic basis at surgery and processed at the Department of Pathology of our institution for diagnostic evaluation. The specimens were fixed in formalin, paraffin-embedded, sectioned at 4 µm, deparaffinized and stained with haematoxylin and eosin, as well as alcian blue, periodic acid–Schiff (after diastase) and colloidal iron to visualize acid-complex sulfated mucins (such as dermatan sulfate and heparan sulfate). Immunohistochemical staining with CD68 antibody was carried out to identify macrophages. In the current study, only these pre-existing slides were re-examined under light microscopy. No additional tissues were collected prospectively.

## Results

### Baseline characteristics of MPS-I patients

A total of 33 patients were included in this study. Five patients were excluded owing to loss of follow-up. The majority were diagnosed with the Hurler phenotype and two with the Hurler–Scheie phenotype. The median age was 13 years (IQR 7.7 to 18.3), with a median follow-up of 12 years (IQR 6.8 to 17.2). Successful HSCT was performed in 33 patients at a median age of 13.37 months (IQR 7.9 to 19.3). Five patients had transplant rejection, for which multiple transplantations were carried out (Table 1).

**Table 1.** Baseline characteristics.

Characteristics		*N* = 33
Sex	Boys, *N* (%)	15 (45.5)
	Girls, *N* (%)	18 (54.5)
Phenotype	Hurler, *N* (%)	31 (93.9)
	Hurler–Scheie, *N* (%)	2 (6.1)
Patient age, median (IQR)		13 years (7.7–18.3)
Follow-up time, median (IQR)		12 years (6.8–17.2)
Mutation trait	Homozygous, *N* (%)	12 (36.4)
	Heterozygous, *N* (%)	21 (63.6)
HSCT*	Transplanted once, *N* (%)	28 (84.8)
	Transplanted twice, *N* (%)	4 (12.1)
	Transplanted three times, *N* (%)	1 (3)
Age at successful HSCT, median (IQR)		13.37 months (7.9–19.3)
IDUA enzyme level after successful HSCT	Normal, *N* (%)	32 (97)
	Below lower level of normal, *N* (%)	1 (3)
Urinary heparan and dermatan sulfate concentration after successful HSCT	Normal, *N* (%)Above upper level of normal, *N* (%)	0 (0)33 (100)

Abbreviations: *N*, number; IQR, interquartile range; HSCT, haematopoietic stem cell transplantation; IDUA, *a*-l-iduronidase.

* Percentages may not sum to 100% due to rounding.

### Examination of the hand of patients with MPS-I and carpal tunnel syndrome

A total of 27 patients (14 boys; 13 girls) were diagnosed with CTS at a median age of 3.64 years (IQR 2.8 to 5.2). Twenty-four patients developed CTS after HSCT and three developed it before HSCT. Twenty-five patients had the Hurler phenotype and two had the Hurler–Scheie phenotype. Sixteen patients had bilateral CTS and 11 had unilateral CTS (five right, six left).

Seven patients experienced symptoms before CTR, with pain and objective muscle weakness being the most frequently observed. Fourteen patients did not experience clinical symptoms before CTR and underwent CTR because of positive signs on electrophysiological tests and/or US. Patients diagnosed with CTS without clinical symptoms showed the highest sensory latency values on electrophysiological tests, whereas patients diagnosed with CTS with clinical symptoms showed the highest motor latency values (Table 2).

**Table 2.** Motor and sensory latency values obtained from electrophysiological testing of the median nerve in mucopolysaccharidosis type I patients diagnosed with CTS with and without the presence of clinical symptoms.

		Motor latency		Sensory latency	
		Right hand	Left hand	Right hand	Left hand
Clinical symptoms	Negative, Median (IQR)	2.90 (2.2–3.7)	2.99 (2.8–3.5)	2.52 (1.9–3.2)	2.89 (2.1–3.9)
	Positive, Median (IQR)	5.15 (2.8–6.3)	4.37 (2.7–9.4)	2.15 (2.1–4.3)	2.46 (2.0–3.2)

Abbreviations: CTS, carpal tunnel syndrome; IQR, interquartile range.

Six patients did not undergo CTR. One patient died before undergoing a CTR and one patient remained on the waiting list. In three patients, no parental consent was provided and in one patient, the risk of anesthesia was too severe. These patients were followed up for a median time of 1.46 years (IQR 0 to 7) after CTS diagnosis. The two patients who did not experience clinical signs or symptoms still had no symptoms of CTS after a mean of 3.17 years.

### Early indicators and risk analyses for development of carpal tunnel syndrome in patients with MPS-I

Correlation analyses between the development of CTS and mean IDUA enzyme level, urinary heparan and dermatan sulfate concentrations and age at successful HSCT showed HRs of 1.481 (95% CI, 0.644 to 3.406; *p* = 0.356), 1.255 (95% CI, 0.720 to 2.188; *p* = 0.423), 0.932 (95% CI, 0.835 to 1.040; *p* = 0.210) and 0.979 (95% CI, 0.923 to 1.038; *p* = 0.478), respectively. These findings were not statistically significant and the large confidence intervals indicated weak predictive power for the influence of CTS development.

**Figure 1.**
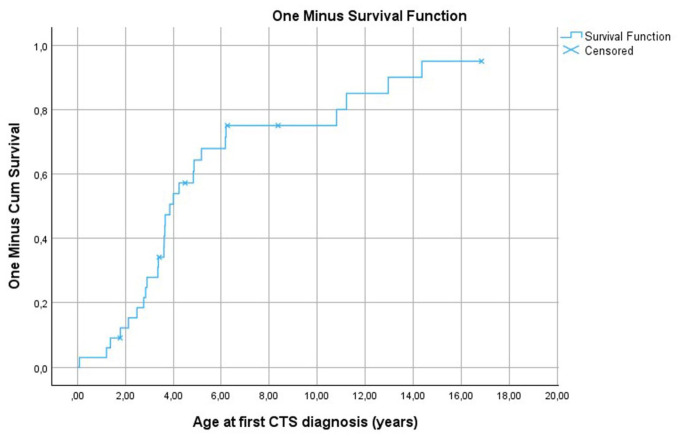
One minus survival function for assessment of cumulative risk for development of carpal tunnel syndrome (CTS) in mucopolysaccharidosis type I patients receiving haematopoietic stem cell transplantation, based on the age in years at the time of first CTS diagnosis. The end of follow-up is portrayed by censored points.

The cumulative risk of developing CTS in this cohort of MPS-I patients was 3, 15, 34, 68 and 80% at 1, 2, 3, 5 years and 10 years of age, respectively. The cumulative risk of developing CTS increased significantly during the first 5–6 years of age and then continued to increase more slowly, ultimately reaching 95% by 15 years of age (Figure 1).

**Figure 2.**
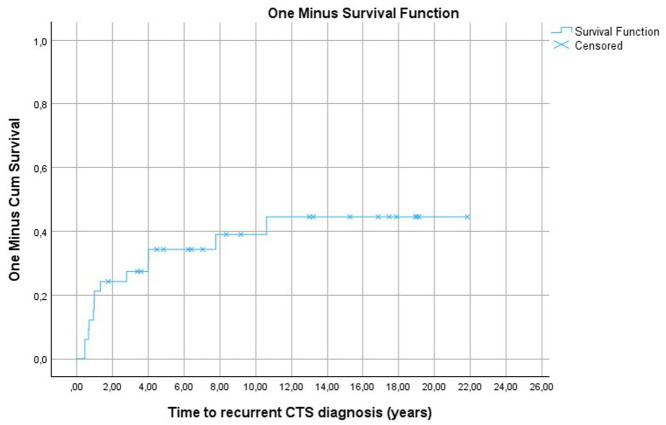
One minus survival function for assessment of cumulative risk for recurrence of carpal tunnel syndrome (CTS) in mucopolysaccharidosis type I patients receiving haematopoietic stem cell transplantation, based on the time from first carpal tunnel release to recurrent CTS diagnosis. The end of follow-up is portrayed by censored points.

The cumulative risk for recurrence of CTS after CTR in this cohort of MPS-I patients was 12%, 24%, 28%, 34%, and 40% at 1, 2, 3, 5 and 10 years after the first CTR, respectively. The cumulative risk increased steeply within the first 2 years after CTR. The risk continued to increase, albeit at a slower rate, ultimately reaching a cumulative risk of 45% by 11 years post-CTR (Figure 2).

### Efficacy of carpal tunnel release in MPS-I patients

Twenty-one patients (12 boys; nine girls) received their first CTR at a median age of 4.29 years (IQR 3.1 to 5.6). Thirteen patients (five boys; eight girls) had a recurrence within a median of 1 year (IQR 0.7 to 4) (Figure 2), with eight patients (three boys; five girls) undergoing repeat CTR. Of the five patients who did not undergo a repeat CTR, four showed mild CTS on electrophysiological tests and/or US and their parents or legal guardians opted against a repeat CTR while one patient remained on the waiting list.

**Figure 3.**
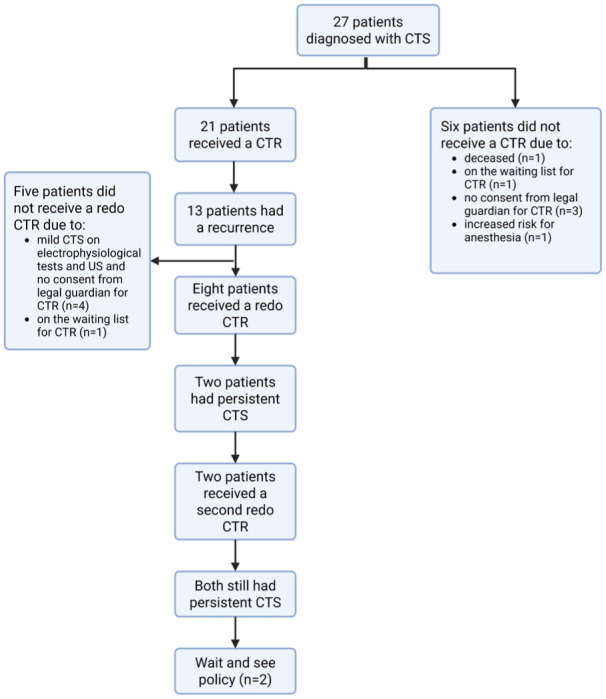
Flow-chart summarizing the clinical course of haematopoietic stem cell transplantation-treated mucopolysaccharidosis type I patients with carpal tunnel syndrome.

After a mean of 4.84 months after repeat CTR, two patients (two girls) had persistent signs of CTS. One patient had bilateral CTS and the other had CTS in the right hand. Both patients underwent a second repeat CTR; the patient with bilateral CTS had persistent CTS in the left hand after 1.6 years and the second patient had persistent CTS in the right hand after 3.2 years. The outcomes of electrophysiological tests and US examinations in these patients oscillated between normal and abnormal values without a clear trend and an expectant policy was decided upon (Figure 3).

Almost all patients showed varying degrees of improvement in both sensory and motor latency values after their first CTR. After repeat CTR, improvements in both sensory and motor latency values were observed but to a lesser extent. Conduction velocity values after the first and repeat CTR improved in most patients but remained below normal values. This indicates persistent median nerve damage. Therefore, there is most likely a persistence of CTS owing to damage to the median nerve, rather than recurrence after repeat CTR. When recurrence/persistence was recorded, the most observed symptoms included tingling sensation, pain, decreased use of hands and muscle weakness.

**Figure 4.**
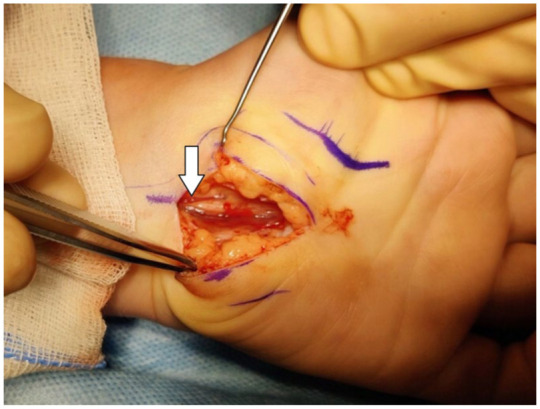
Intra-operative photograph of the median nerve with its distinct ‘hourglass’ deformity (arrow).

The intraoperative findings during CTR included a thickened median nerve, often with a distinct ‘hourglass’ deformity and varying degrees of vascular injection and discolouration, ranging from pale to hyperaemic (Figure 4). The flexor retinaculum was thickened and synovial tissues appeared swollen in six cases.

### Histological analysis of flexor retinaculum tissue

**Figure 5.**
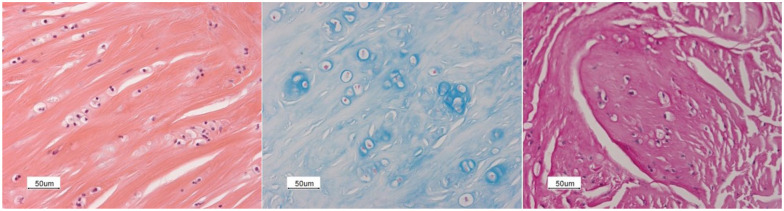
Light microscopy of the synovium from the carpal tunnel collected 10.1 years after HSCT of a Hurler patient treated with haematopoietic stem cell transplantation at 14.6 months. Left: haematoxylin and eosin stain showing an abundant number of foam cells in between collagen-like type fibers. Middle: Alcian blue stain showing an abundant number of foam cells with positively stained material inside the cell. Right: Periodic acid-Schiff after diastase stain showing an abundant number of foam cells with positively stained material inside the cell.

To assess the effect of HSCT on the resolution of degradation product accumulation, flexor retinaculum tissue samples were re-examined. These samples revealed a large number of cells with abnormally large, vacuolated cytoplasm in eight of 11 patients after a median of 7.31 years (IQR 2.3 to 10.5) post-HSCT. No inflammation was observed owing to the lack of cell influx. The cytoplasm of these ‘foam cells’ stained positive for Alcian blue (middle), colloidal iron (not shown) and periodic acid-Schiff with diastase (right) (Figure 5). No foam cells were observed in tissue samples from three patients after a mean of 2 years post-HSCT. These three patients had the Hurler phenotype, received successful HSCT at a mean age of 19.31 months and developed CTS at a mean age of 3.3 years.

## Discussion

Despite successful HSCT, CTS remains one of the most common hand disorders in patients with MPS-I (Viskochil et al., 2017). This study has reported a high prevalence of CTS and the highest risk of developing CTS is within the first 6 years of life. Seven patients expressed clinical symptoms before CTR and a high recurrence rate was observed, with the highest risk within the first 2 years after surgery.

The recurrence rate of CTS after CTR in this study was three-fold higher than that in previous reports (Thirache et al., 2022; Viskochil et al., 2017). This difference may be explained by differences in the data acquisition strategy. The structured longitudinal follow-up implemented at our specialist hospital contrasts with registry studies that rely on active reporting of data. Systematic screening may uncover more recurrent CTS events and allow for a better estimate of the true recurrence risk. Previous reports have shown a higher recurrence rate of CTS in MPS-I patients with more attenuated phenotypes treated with ERT, compared with those with more severe phenotypes, treated with HSCT (Thirache et al., 2022; Viskochil et al., 2017). However, since most patients in this study had the severe phenotype and only a few had the attenuated phenotype, the difference in recurrence rates between these groups could not be statistically confirmed. An explanatory hypothesis for the high recurrence rate includes a narrower carpal tunnel owing to dysostotic abnormalities of the bones forming the borders of the carpal tunnel. However, data on carpal tunnel diameters in children with MPS-I compared with their healthy peers are not available. Data on flexor tendon diameters in patients with MPS I are not available. These children often develop trigger fingers and thicker tendons owing to GAG accumulation which might contribute to median nerve compression in the carpal tunnel and the high recurrence rate after surgery. Further investigation of the mechanisms underlying this high recurrence rate could improve our understanding of CTS pathology in patients with MPS-I and guide future management strategies.

The clinical presentation of CTS in MPS-I patients differs from that in the non-MPS population (Aldenhoven et al., 2009; Dabaj et al., 2020; Haddad et al., 1997; Patel et al., 2020; van Meir and de Smet, 2003; Wraith and Alani, 1990; Yuen et al., 2007). Pain and objective muscle weakness predominate over classical symptoms such as tingling sensations, numbness and nocturnal discomfort. Most patients do not exhibit any clinical symptoms. This lack of symptom expression is hypothesized to be partially due to chronic compression and the gradual onset of symptoms. This results in MPS-I patients adapting and failing to report symptoms unless motor dysfunction becomes prominent. In patients with more severe MPS-I phenotypes, cognitive impairment may further delay recognition or expression of symptoms (Clarke, 2024; Dabaj et al., 2020). However, once motor dysfunction becomes sufficiently pronounced, symptoms are typically reported either by the patient or by their parents who observe functional decline. Our findings are consistent with this hypothesis, as patients who reported clinical symptoms of CTS tended to show higher motor latency values. Despite the high prevalence of CTS found in this cohort (82%) and other studies (67–96%), it is important to be aware of the frequent lack of clinical symptoms (Guffon et al., 2021; Schmidt et al., 2016; Souillet et al., 2003; van Heest et al., 1998).

Earlier studies have noted that CTS in MPS-I often presents atypically (Aldenhoven et al., 2009; Dabaj et al., 2020; Haddad et al., 1997; Patel et al., 2020; van Meir and de Smet, 2003; Wraith and Alani, 1990; Yuen et al., 2007). By demonstrating that 82% of our patients met electrophysiological and/or US criteria for CTS, while most did not report clear clinical symptoms, our study confirms that the diagnosis of CTS in MPS-I patients relies primarily on electrophysiological tests and/or US examinations. As highlighted in this study, the risk of developing CTS is highest during the first 6 years of life, a critical period when a child’s hand function is still developing. This underscores the importance of accurate diagnostic tools to support hand function development, prevent irreversible nerve damage, improve surgical outcomes and enable better assessment of therapeutic interventions and their impact on CTS development (Viskochil et al., 2017; White et al., 2010).

For decades, if clinical symptoms are not apparent, electrophysiological testing has been the reference standard for diagnosing CTS (Clarke, 2024; Muenzer et al., 2009). In recent years, US examination has become a more patient-friendly method for the diagnosis of CTS. However, for both strategies, reference values for paediatric patients are based on small sample sizes and may not fully account for the altered connective tissue characteristics and musculoskeletal anatomy of MPS-I, which could influence diagnostic accuracy. Therefore, they should be used as guidance rather than strict cutoff values for the diagnosis of CTS (Cartwright et al., 2012; Ryan et al., 2019). In addition, in our cohort, latency values did not show complete improvement 1 year after primary CTR. After a repeat CTR, these improvements were even smaller. Several factors may contribute to this incomplete electrophysiological recovery in MPS-I patients. An important consideration is the possibility of irreversible axonal damage (Caetano, 2003). Chronic compression of the median nerve can induce axonal degeneration and in advanced disease, this neural injury may not be fully reversible even after surgical decompression, underscoring the importance of early diagnosis and timely surgical intervention (Aulisa et al., 1998; Padua et al., 1996). Another possible explanation is suboptimal surgical decompression owing to abnormal tissue consistency or anatomical variation, which increases the risk of residual compression persisting even after surgery (Jones et al., 2012). Furthermore, the progression of systemic diseases poses a significant challenge in this population. Despite surgical intervention, the continuous deposition of storage material and connective tissue remodelling characteristics of MPS-I may lead to renewed or sustained median nerve impairment over time, preventing full electrophysiological recovery even in long-term follow-up. It is also important to consider the role of follow-up duration when interpreting electrophysiological recovery, as studies in non-MPS adults have shown incomplete normalization in all advanced cases of CTS, while most mild and moderate cases of CTS showed complete normalization of electrophysiological values after a follow-up period of 6 months (Aulisa et al., 1998; Padua et al., 1996). A later study analysing electrophysiological recovery after CTR for advanced CTS found that some patients exhibited further improvement in electrophysiological measures up to 2 years after surgery (Kanatani et al., 2013). These data from non-MPS populations align with our finding that electrophysiological values in MPS-I often do not reach complete normalization 1 year after CTR, suggesting that both the severity of preoperative axonal damage and the prolonged time course of recovery contribute to persistent abnormalities. This highlights the importance of an adequate follow-up duration, as a follow-up that is too short could cause false-positive diagnoses of persistent or recurrent CTS. Nonetheless, greater baseline axonal damage remains a key predictor of incomplete normalization and this may be even more relevant in MPS-I. Our findings highlight the requirement for early detection and timely intervention to minimize irreversible nerve injury, as persistent abnormalities after CTR should prompt consideration of axonal damage, technical adequacy of decompression, and the ongoing effects of the underlying metabolic disorder.

**Figure 6.**
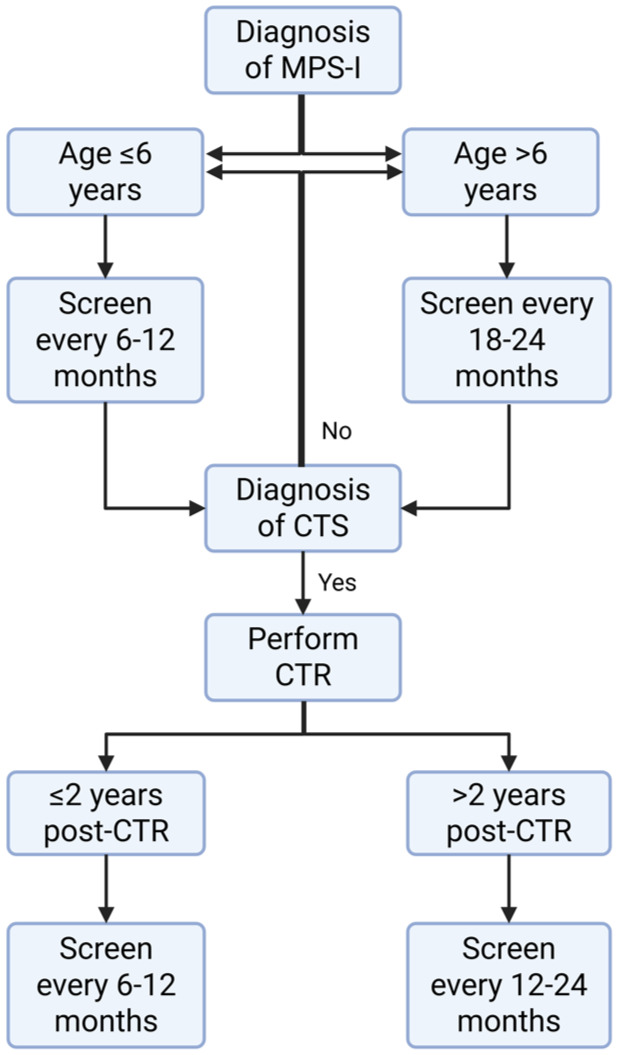
Flow chart for the screening of carpal tunnel syndrome using electrophysiological tests pre- and post-carpal tunnel release (CTR) in MPS-I patients based on age and time post-CTR. Created with BioRender.com.

Because of the high prevalence of CTS with often minimal clinical symptoms, preventive CTR is a justifiable option. However, in our opinion, preventive CTR is not indicated if these patients are systematically screened at fixed time points (Figure 6). Based on our findings, a significantly increased risk of CTS development within the first 6 years of age was observed. Accordingly, we propose that patients with MPS-I be screened for CTS using electrophysiological tests or US at least annually, preferably every 6 months, during this initial period. After these first 6 years, the frequency of screening could be decreased to once every 18 months to 2 years to decrease the burden on these patients and hospital costs. In addition, our study shows that after CTR, a significant increase in the risk of recurrence was observed within the first 2 years. Therefore, we propose that MPS-I patients be screened every 6 months during the initial period after CTR. After this period, screening can be decreased to once every year or every other year.

This study shows that HSCT is not effective in preventing or curing CTS. This is in line with previous registry-based and cohort studies that found a substantial CTS burden in MPS-I patients despite successful HSCT and/or ERT (Dabaj et al., 2020; Guffon et al., 1998; Khanna et al., 2007; Souillet et al., 2003; Viskochil et al., 2017; White et al., 2010; Wyffels et al., 2017). Additionally, this study did not observe significant correlations between the predictors and the outcomes of CTS. The wide confidence intervals and lack of statistically significant hazard ratios are probably explained by the limited sample size inherent to rare diseases and restricted variability in the biochemical predictors after HSCT, which reduces statistical power and increases uncertainty in the regression estimates. As mentioned previously, the pathophysiology of CTS in patients with MPS-I remains uncertain. Unfortunately, we were unable to determine the primary mechanism underlying CTS susceptibility in this study. Possible aetiological factors include a nerve that is more susceptible to compression, abnormal carpal tunnel anatomy, reduced tunnel space from the accumulation of GAGs in the tendons and altered mechanical properties of the flexor retinaculum owing to changes in collagen and elastin structure or a combination of these factors (Hinek and Wilson, 2000; Lau et al., 2024). However, some patients’ flexor retinaculum tissue samples did not show foam cells after HSCT and despite this absence, our clinical data indicate that such biochemical and histological improvements are insufficient to prevent median nerve compression, supporting the concept that residual connective tissue and bony abnormalities continue to drive CTS pathology in MPS-I. Additionally, as histological analyses were limited to tissue samples obtained on an opportunistic basis during clinically indicated procedures, selection bias cannot be excluded from this study. Therefore, the findings may not fully represent the histopathological variability across the broader MPS-I population.

The main limitation of this study was its retrospective nature. However, since a relatively large cohort of MPS-I patients was systematically screened regularly, a large amount of data on symptoms, signs and diagnoses was available. These patients had a long follow-up period and therefore, the development of CTS and recurrence could be assessed, preventing false negatives. Future research should involve prospective, longitudinal studies with predefined outcome measures comparing surgical intervention with conservative and/or medical management such as neuro-protective agents, splinting or physiotherapy. Such studies could provide valuable insights to better define the optimal treatment plan for primary and recurrent CTS in patients with MPS-I. Advanced imaging techniques such as high-resolution US and dynamic MRI, with detailed morphometric analysis of the carpal tunnel, flexor tendons and flexor retinaculum, may further elucidate the underlying pathophysiological mechanisms of CTS development and help explain the observed differences in recurrence rates between severe and attenuated phenotypes. Finally, as emerging systemic treatments, such as gene therapy, show promise in preventing or delaying the development of CTS (Tucci et al., 2026) and become more widely implemented, future studies should examine their long-term impact on the incidence, progression and postoperative outcomes of CTS.

In conclusion, this study emphasizes the high prevalence and complex nature of CTS in patients with MPS-I. An atypical clinical presentation, proven high recurrence rate and abnormal histopathological findings underscore the challenges of managing this disorder.
